# Transcranial direct current stimulation of the posterior parietal cortex biases human hand choice

**DOI:** 10.1038/s41598-020-80611-8

**Published:** 2021-01-08

**Authors:** Kento Hirayama, Takayuki Koga, Toru Takahashi, Rieko Osu

**Affiliations:** 1 Graduate School of Human Sciences, Waseda University, Tokorozawa, Saitama Japan; 2 Faculty of Human Sciences, Waseda University, 2-579-15 Mikajima, Tokorozawa, Saitama Japan

**Keywords:** Decision, Decision

## Abstract

Hand choices—deciding which hand to use to reach for targets—represent continuous, daily, unconscious decisions. The posterior parietal cortex (PPC) contralateral to the selected hand is activated during a hand-choice task, and disruption of left PPC activity with a single-pulse transcranial magnetic stimulation prior to the execution of the motion suppresses the choice to use the right hand but not vice versa. These findings imply the involvement of either bilateral or left PPC in hand choice. To determine whether the effects of PPC’s activity are essential and/or symmetrical in hand choice, we increased or decreased PPC excitability in 16 healthy participants using transcranial direct current stimulation (tDCS; 10 min, 2 mA, 5 × 7 cm) and examined its online and residual effects on hand-choice probability and reaction time. After the right PPC was stimulated with an anode and the left PPC with a cathode, the probability of left-hand choice significantly increased and reaction time significantly decreased. However, no significant changes were observed with the stimulation of the right PPC with a cathode and the left PPC with an anode. These findings, thus, reveal the asymmetry of PPC-mediated regulation in hand choice.

## Introduction

Flexible adaptation to the outside world mandates appropriate action selection. Hand choice—deciding which hand to use to reach for targets—is an example of a daily unconscious action selection. Previous studies have shown that various factors are related to hand choice, such as handedness^1,2^, hand-use history (whether the used hand successfully completed the tasks or not)^3^, and physical position relative to the target (the probability of right-hand use increases when the targets are situated on the right side of the body)^4–7^.

Kalaska et al. reported that the posterior parietal cortex (PPC) and premotor area (PM) are related to action selection^8^. PPC plays various roles in the motor domain, such as action planning, motor imagery, gesture recognition, and tool use. It is also involved in more cognitive functions, such as spatial attention and working memory to guide perception, decisions, and behaviour^9,10^. Specifically, PPC encodes action plans based on the information received from the various brain areas, such as the visual cortex, basal ganglia and premotor area. A non-human primate study has shown that saccade and reach plans are encoded in parallel in different areas of PPC during the performance of the experiment in which monkeys were required to select either making a saccade to a target or reaching for a target with their hands^11^. The study argued that PPC accumulates sensory information to evaluate the appropriate action selection and, thus, plays a critical role in this process. Fitzpatrick et al. reported that while functional magnetic resonance imaging (fMRI) revealed bilateral increases in PPC activity during hand-choice tasks, this increase is enhanced in the PPC contralateral to the selected hand^12^. The investigators further posited that their results support the Posterior Parietal Interhemispheric Competition (PPIC) model, which states that both PPC encode hand-specific actions, and activity in and across both the hemispheres competes for the selection. However, Oliveira et al. showed that the right-hand choice was suppressed following the disruption of the left PPC with single-pulse transcranial magnetic stimulation (TMS) just prior to the execution of the reach, but not vice versa^4^, thus, indicating more dominant involvement of the left PPC in hand choice than the right PPC. Based on these conflicting results, whether PPC involvement in hand choice is symmetrical or asymmetrical remains unclear. The primary objective of the present study was to determine whether the effect of PPC on hand-choice is bilateral or left-dominant using transcranial direct current stimulation (tDCS).

The secondary purpose of this study was to verify the causal relationship between PPC excitability and hand choice by increasing or decreasing the excitability of bilateral PPCs using tDCS. While Fitzpatrick et al. showed that fMRI activation correlated with hand choice^12^, this observation does not imply that the area is causally involved in the choice itself. Oliveira et al., in contrast, demonstrated that the left PPC is involved in the neural process of hand selection immediately before its execution^4^. While single-pulse TMS provided causal evidence by disrupting an on-going neuronal process, it did not convey whether neuromodulatory changes in PPC excitability caused by plasticity such as long-term potentiation (LTP) or depression (LTD) affect the decision regarding hand choice. It, therefore, remains unclear whether PPC excitability in itself is essential to hand choice.

In recent years, tDCS has garnered attention as a tool to investigate a causal link between the behaviour and the neuronal activity of stimulated brain areas in neuroscience^13^. Anodal and cathodal stimulation of the motor cortex using tDCS can increase and decrease the cortical excitability, respectively^14^, for up to 90 min^15^. In this study, we increased or decreased the cortical excitability of PPC using tDCS and examined its online and residual effects on the hand-choice probability and choice reaction time.

If both PPCs are symmetrically involved in hand choice, we can expect the opposite effects when the polarity of stimulation is reversed between the left and right PPCs. To test this hypothesis, we stimulated the left PPC with a cathode and the right PPC with an anode, or vice versa. If the PPC’s excitability plays a causal role in hand choice, the neuromodulation will have a residual effect on hand choice during the post-stimulation period. Therefore, we tested the hand choice before, during, and after the stimulation.

## Methods

### Participants

This study included 16 right-handed healthy participants (six females; overall mean age, 21.3 ± 1.3 years) who provided written informed consent for participation and were remunerated for their participation. The required sample size was calculated using G*Power v3.1 with power of 0.9, alpha level of 0.05, and effect size of 0.4. Following the power analysis, a sample size of 16 was required for a two-way repeated measures analysis of variance (ANOVA) with three measurements. The large effect size was determined based on previous studies that examined the effects of tDCS on decision-making^16–18^. The inclusion criteria were as follows: (1) no history of nerve injury or orthopaedic injury to the upper extremity; (2) no history of epilepsy, seizures, brain damage, head injury, loss of consciousness, or brain surgery with implants in the head; (3) no history of chronic or acute neurological, psychiatric, or medical illnesses; (4) no history of drug addiction or tobacco consumption; and (5) not currently pregnant. Additionally, caffeine was not consumed within 12 h prior to the start of the experiment. All procedures were approved by the Waseda University Ethics Committee and were performed in accordance with the Declaration of Helsinki.

### Experimental design

#### Experimental setup

**Figure 1:**
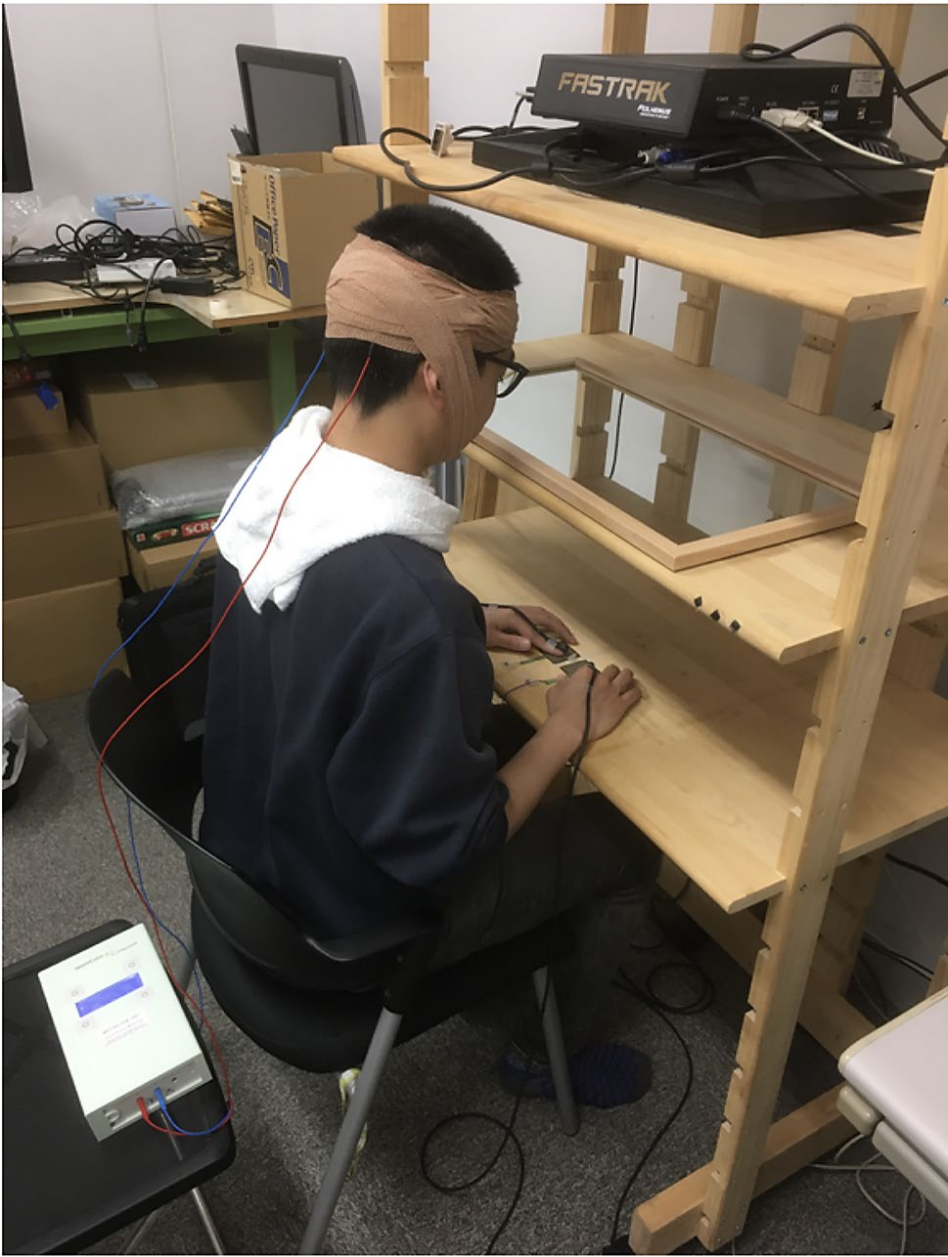
Experimental setup. A display was installed facing downward on a shelf situated above the participant’s visual field. The picture was projected on a mirror placed halfway between the display and the table surface. By viewing the stimuli on a mirrored surface, the participants could gain the impression that the stimuli were on the same plane as their hands. The position of the hands was indicated by two black dots on the reflected display; these points corresponded to the position of three-dimensional motion-tracking sensors worn on both index fingers by the participant.

**Figure 2:**
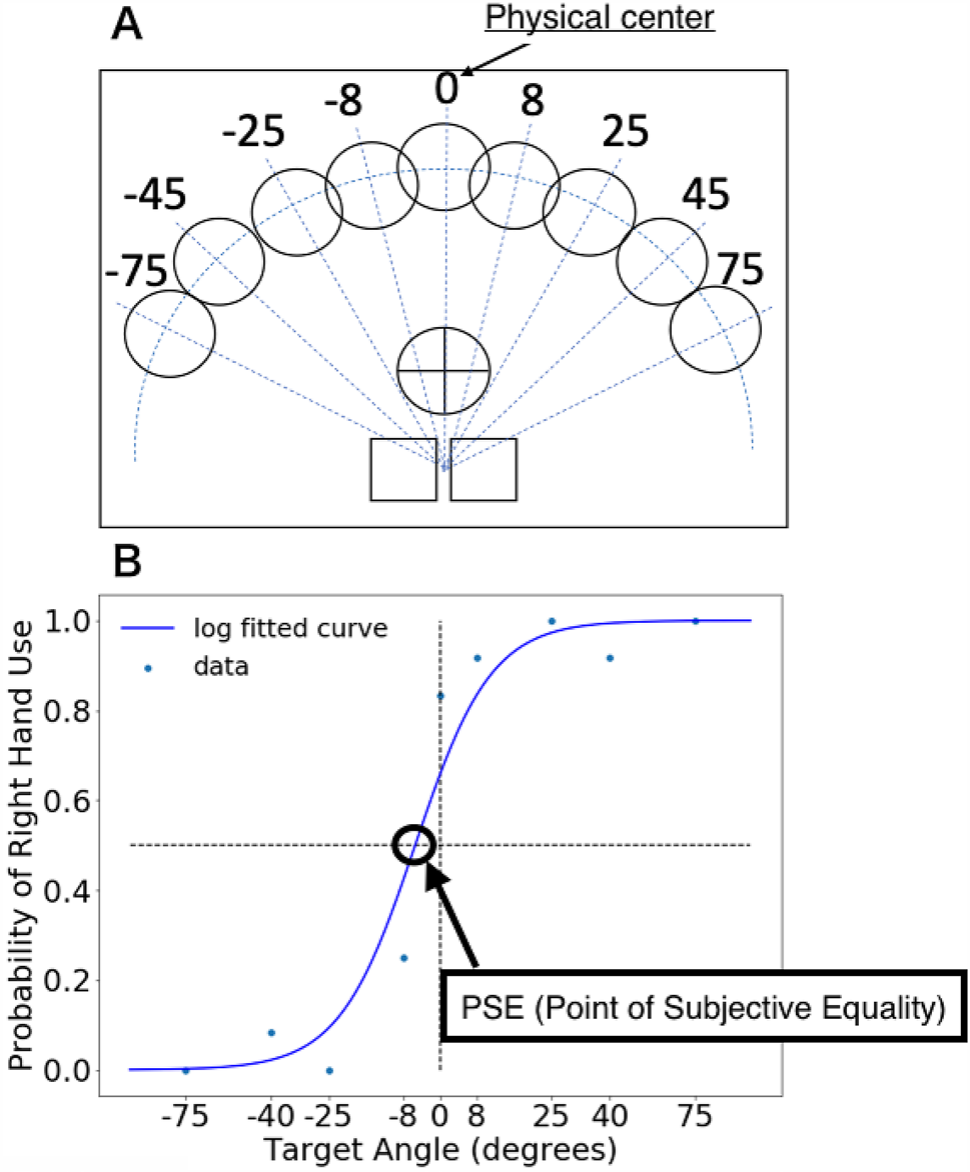
Target positions and point of subjective equality of hand choice. (**A**) Target positions. Starting position (bottom two squares), fixation point (central “+” mark), and the nine possible targets. The targets were set at angles of 0°, 8°, 25°, 45°, and 75° to either side of the mirror’s midline. (**B**) Point of subjective equality (PSE). The probability of choosing the right hand for each target is fitted to a logistic function for each participant. The horizontal axis indicates the target angle, where 0 is the midline as the physical centre (positive degree means rightward rotation and negative degree means leftward rotation relative to the midline). The vertical axis indicates the rate of right-hand selection. PSE, the estimated location at which participants were equally likely to use the right and left hand, was thus determined.

The participants sat in a chair with their hands comfortably positioned on the surface of a table. A display was placed horizontally above the table and a mirror was placed halfway between the display and the table surface. The heights at which they were placed ensured that the participants could see the mirror but not the display or the hands. The presentation of the stimuli on the display and their reflection by the mirror yielded the impression that the stimuli were presented on the table (Fig. 1). Two three-dimensional (3D) motion-tracking sensors (Fastrak, Polhemus, Colchester, VT, USA) were attached to the index fingers of each participant. The position of each fingertip was measured at a sampling rate of 60 Hz. Feedback in the form of two black dots indicating the current position of both hands were presented on the display. The participants were instructed to place their fingertips on the pressure sensors on the table so that the sensors could detect the hand that the participants released from the table. A “+” symbol was displayed within a circle positioned 2.5 cm away from the central position to signal the target for visual fixation location (Fig. 2A).

#### Task protocol

**Figure 3:**
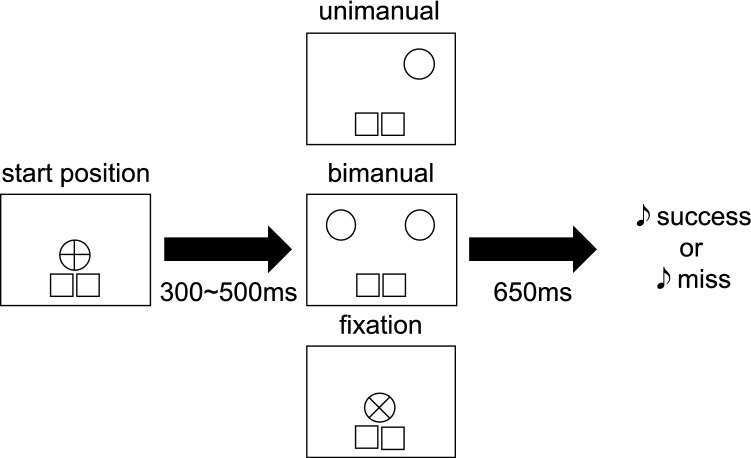
Task protocol. At the start of each trial, the participants were instructed to press the pressure sensors and focus their eyes on the fixation point. Three types of trials were thereafter presented randomly. In “unimanual reach trials”, the participants reached with one hand toward the target. In “bimanual reach trials”, two target circles were presented and the participant simultaneously reached out to them with both hands. In “fixation catch trials”, the “+” at the centre of the fixation circle changed to “×” and the participants moved both hands to the fixation circle. When one of the sensors on the index finger entered the target circle within 650 ms, the target disappeared and a sound indicating success was played. Otherwise, the target did not disappear and a sound signalling failure was played.

At the start of each trial, the participants were instructed to press the pressure sensors and keep their eyes focused on the fixation point (Fig. 3). One of three types of trials was then started with variable inter-trial intervals of 300–500 ms. In “unimanual reach trial”, a 4-cm-diameter target circle was presented at one of nine positions on a semicircle situated approximately 27 cm away from the start position. The targets were presented each of nine positions in a random order. The participants were instructed to reach the target with either hand as quickly as possible within 650 ms of target presentation. The unused hand was required to be retained at the initial position. When a sensor on the index finger was placed within the radius of the target within 650 ms, the target disappeared and a sound indicating success was played. If a sensor was not placed in the target radius within 650 ms, the target did not disappear and a sound indicating failure was played. In “bimanual reach trial”, two targets were presented on a semicircle simultaneously and the participants were instructed to reach both targets simultaneously with two hands within 650 ms. This trial was incorporated to reduce the probability that the participants would always use the same hand. In “fixation reach trial”, the “+” mark on the fixation point changed to an “×” mark. The participants were instructed to place both index fingers within the fixation circle within 650 ms. This trial was incorporated to confirm that the participant engaged in fixation before target presentation. The participants were permitted to move their eyes freely after the target was presented. The participants were instructed to return their hand to the start position after they heard the feedback sound.

#### Measurements

The selected hand was defined as the hand that reached a distance 2.5 cm away from the start position before the other hand. To measure the hand choice, we calculated the participant’s probability of choosing the right hand for each target. Reaction time (RT) was defined as the duration between target presentation and the release of the selected hand from the pressure sensor.

### Transcranial direct current stimulation protocol

**Figure 4:**
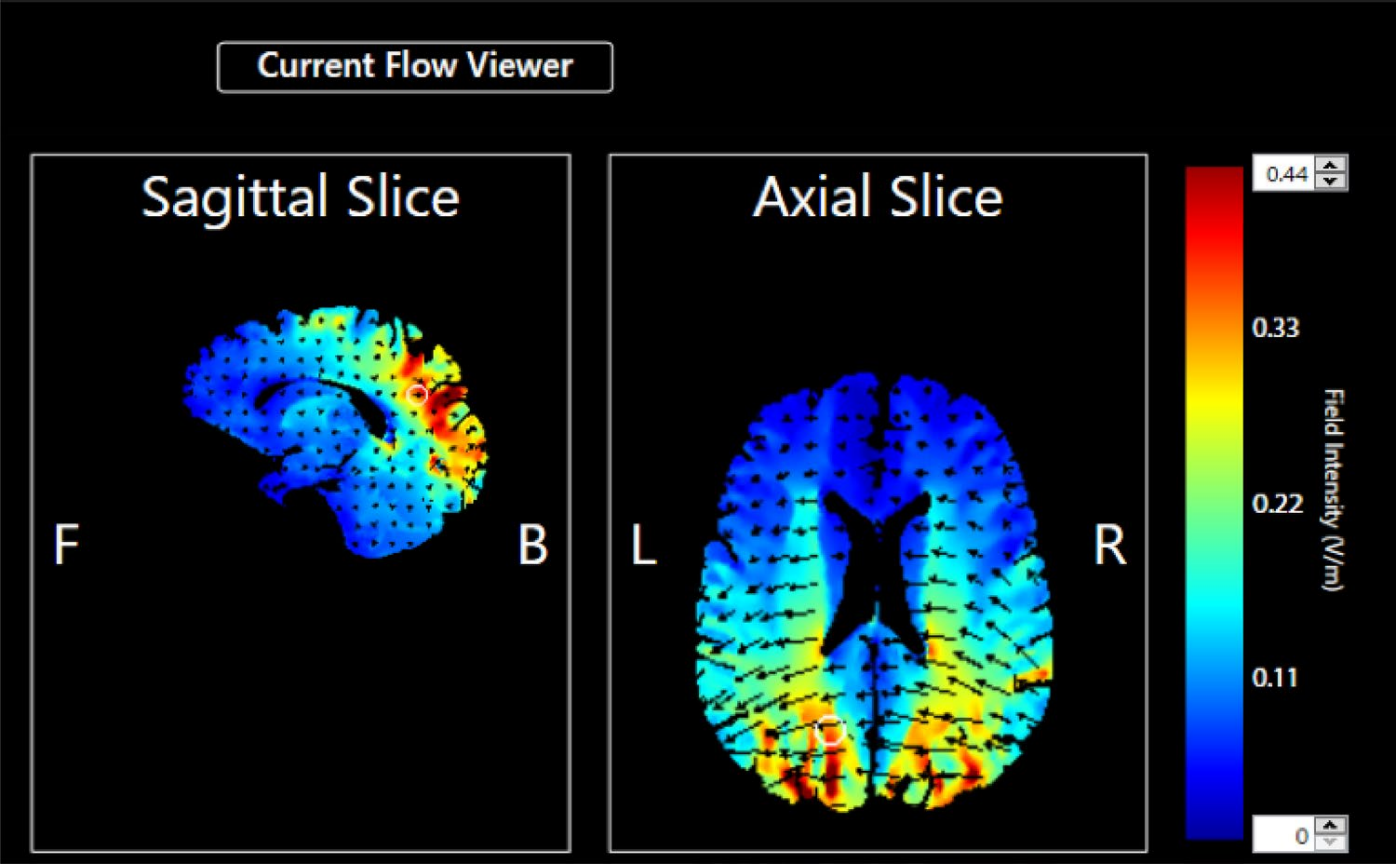
The result of cortical current flow simulation of transcranial direct current stimulation (tDCS) in the present experiment. The cathodal and anodal electrodes were assigned either P3 (left posterior parietal cortex, PPC) or P4 (right PPC). The stimulation induced an electric field of 0.4 V/m over both PPCs.

Direct electrical current was delivered using a neuroConn DC Brain Stimulator Plus unit (Rogue Resolutions, Wales, UK) featuring rubber electrodes placed in saline-soaked sponge pads. The size of the electrode pad was 5 cm × 7 cm. Bilateral montage was selected. To stimulate the posterior parietal cortex, each electrode was placed at P3 and P4 according to the International 10–20 System for electroencephalogram (EEG) electrode placement. Two stimulation conditions were assigned: (1) the left PPC was stimulated with a cathode and the right PPC with an anode (LCRA condition), and (2) the left PPC was stimulated with an anode and the right PPC with a cathode (LARC condition). A constant current of 2 mA (current density of 0.06 mA/cm^2^) was applied for a total of 10 min with additional 15 s of ramp up and ramp down at the beginning and end of the stimulation, respectively. The simulation of cortical current flows using tDCS Explore^TM^ (Soterix Medical, New York, NY, USA) showed that the stimulation achieved an electrical field strength of approximately 0.4 V/m on both PPCs (Fig. 4).

### Procedure

**Figure 5:**
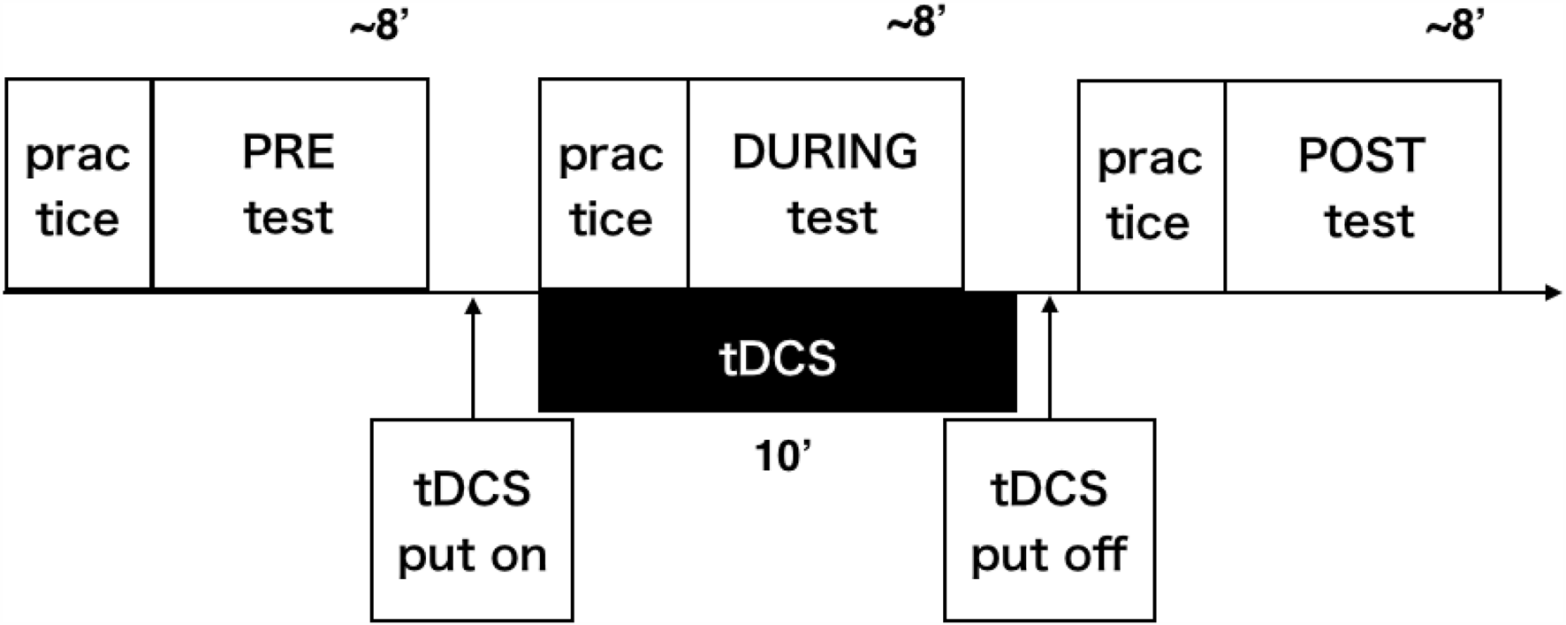
Procedure. Half of the participants underwent stimulation under the left cathode and right anode condition on the first day, while the other half underwent the left anode and right cathode condition; the conditions were switched on the second day one week after the first day. One test block consisted of 120 reach trials. Participants performed two test blocks before (the PRE phase), during (DURING phase), and after (POST phase) the stimulation. Before each phase, the participants completed practice sessions of 60 trials. Transcranial direct current stimulation was applied for 10 min from the start of the practice session in the DURING phase. Each combined practice and test phase took approximately 8 min.

An overview of the procedure is presented in Fig. 5. One block consisted of 108 unimanual reach trials (nine targets on the semicircle displayed 12 times), six bimanual reach trials, and six fixation reach trials presented in a pseudo-random order. The participants performed two blocks before (PRE), during (DURING), and after (POST) the stimulation. Before the commencement of the PRE, DURING, and POST phases, the participants completed three practice sessions of 20 trials. The three practice sessions consisted of two types of predetermined hand-choice sessions (only the right and left hand) in a random order followed by the free choice session. We counter-balanced the order of the stimulus conditions in each participant, i.e. half of the participants underwent LCRA on the first day and LARC on the second day with an interval of one week, and vice versa.

### Statistical analysis

#### Probability of hand choice

By fitting a logistic function to the choice probability data, we determined the point of subjective equality (PSE), i.e. the location at which the participants were estimated to be equally likely to use either the right or left hand (Fig. 2B). PSE in the PRE, DURING, and POST stimulation phases were compared for each stimulation condition using a two-way repeated-measures ANOVA across the stimulation phases (PRE, DURING, and POST) and stimulation conditions (LCRA and LARC). Subsequently, post hoc paired-sample multiple *t* tests with Bonferroni correction were performed to compare PSE of each stimulation phase and condition.

#### RT

The median RT was determined for each target location. We averaged the median RTs for two targets around the individual PSE, which reflected targets at the most ambiguous locations where hand choice competition was the greatest. Since a learning effect was expected to improve the RTs, the data was divided into two groups based on whether they were recorded on the first or second day (Day 1 and Day 2, respectively) for statistical analysis. A two-way ANOVA across the stimulation phases (PRE, DURING, and POST) as intra-participant and stimulation conditions (LCRA and LARC) as inter-participant tests were performed for each day (Day 1 and Day 2). Subsequently, post hoc paired-sample multiple *t* tests were performed with Bonferroni correction.

Before each statistical analysis, normality of data was confirmed using the Shapiro–Wilk test. Spherical assumption was not rejected using Mauchly’s spherical test. Furthermore, the homogeneity of variance between the participants was confirmed using the Levene test. The analyses were performed using SPSS v26 (IBM Inc., Armonk, NY, USA).

## Results

### Probability of hand choice

**Figure 6:**
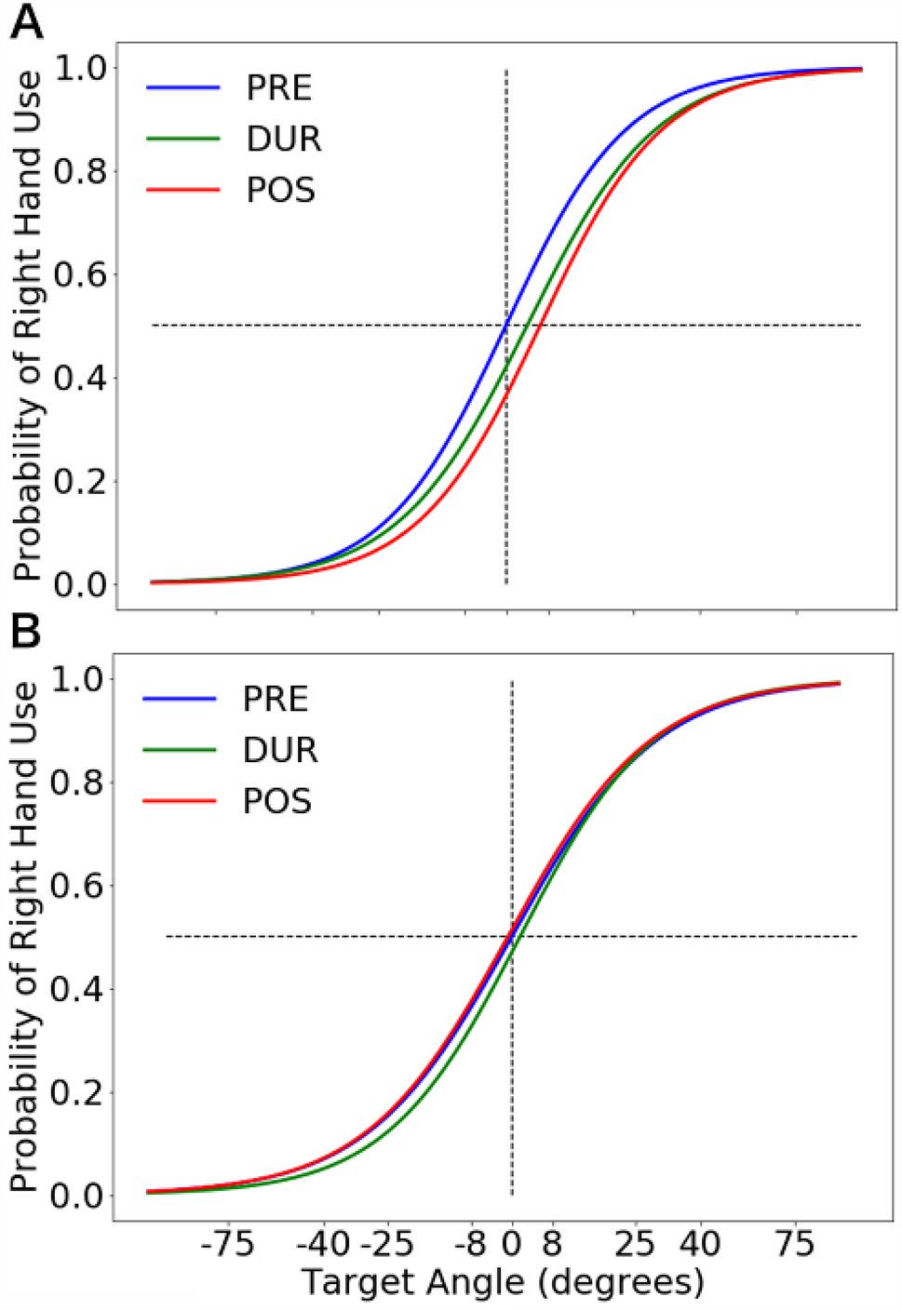
Logistic function was fitted to the probability of right-hand choice and averaged across the participants in the left cathode and right anode stimulation condition (**A**) and the left anode and right cathode stimulation condition (**B**). Blue-line, PRE phase; green-line, DURING phase; red-line, POST phase.

**Figure 7:**
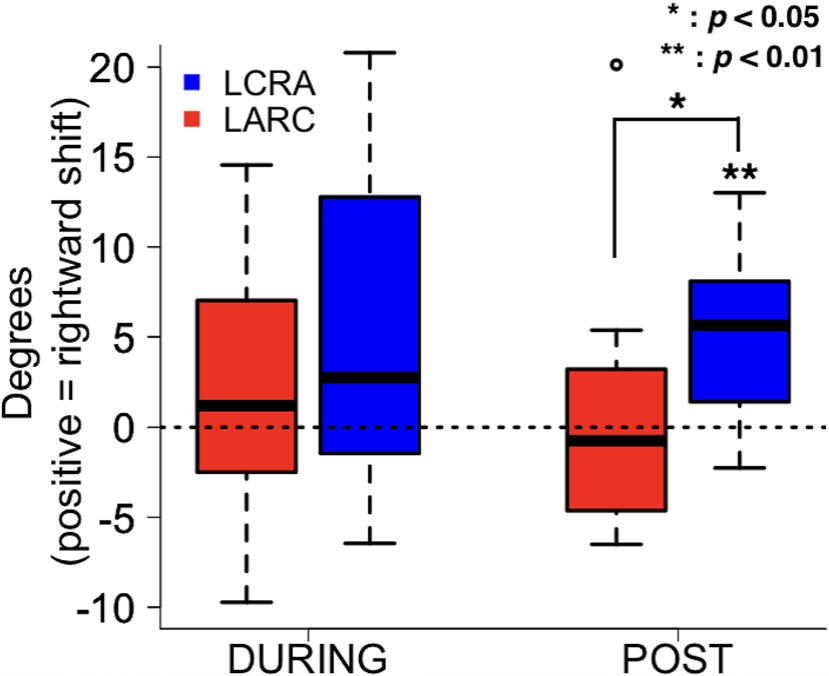
The change in point of subjective equality (PSE) relative to the PRE phase in each stimulation phase. Blue box, left cathode and right anode stimulation condition; Red box, left anode and right cathode stimulation condition. The horizontal line inside each box represents the median, the boxes extend to the lower and upper quartiles, and the whiskers extend to the extreme values other than outliers. Positive values indicate a rightward shift in PSE, i.e. the left hand was used more frequently than the right hand relative to their frequencies of use in the PRE phase.

A logistic function was fitted to the probability of right-hand choice and averaged across participants under the LCRA (Fig. 6A) and LARC conditions (Fig. 6B). In the LCRA condition (Fig. 6A), the logistic function considerably moved toward the right in favour of the left-hand choice during and after the stimulation relative to before stimulation. In contrast, in the LARC condition (Fig. 6B), the logistic function did not significantly move during and after the stimulation. The effect of tDCS on hand choice was evaluated using PSE (Fig. 7). A two-way repeated ANOVA demonstrated a significant interaction between the stimulation phases and conditions (*F* (2, 28) = 4.053, *p* = 0.028, *η*^2^_*p*_ = 0.224) and a main effect of the stimulation phases (*F* (2, 28) = 3.449, *p* = 0.046, *η*^2^_*p*_ = 0.198). The main effect of the stimulation conditions (*F* (1, 14) = 2.932, *p* = 0.109) was not significant. The simple main effect of the stimulation phase under the LCRA condition (*F* (2, 28) = 6.181, *p* = 0.006, *η*^2^_*p*_ = 0.306) was significant. Paired-sample multiple *t* tests corrected for multiple comparisons (Bonferroni correction) demonstrated that PSE moved significantly to the right in favour of the left-hand choice after stimulation relative to before the stimulation (*adj p* = 0.005, *d* = 1.15). The change in PSE was significantly larger under the LCRA condition relative to the LARC condition (*adj p* = 0.040, *d* = 0.89) after the stimulation. In contrast, the simple main effect of stimulation phase was not significant under the LARC condition (*F* (2, 28) = 1.031, *p* = 0.37). These results indicate that PSE shifts to the right and increases the probability of left-hand choice under the LCRA stimulation condition but not vice versa.

### RT

**Figure 8:**
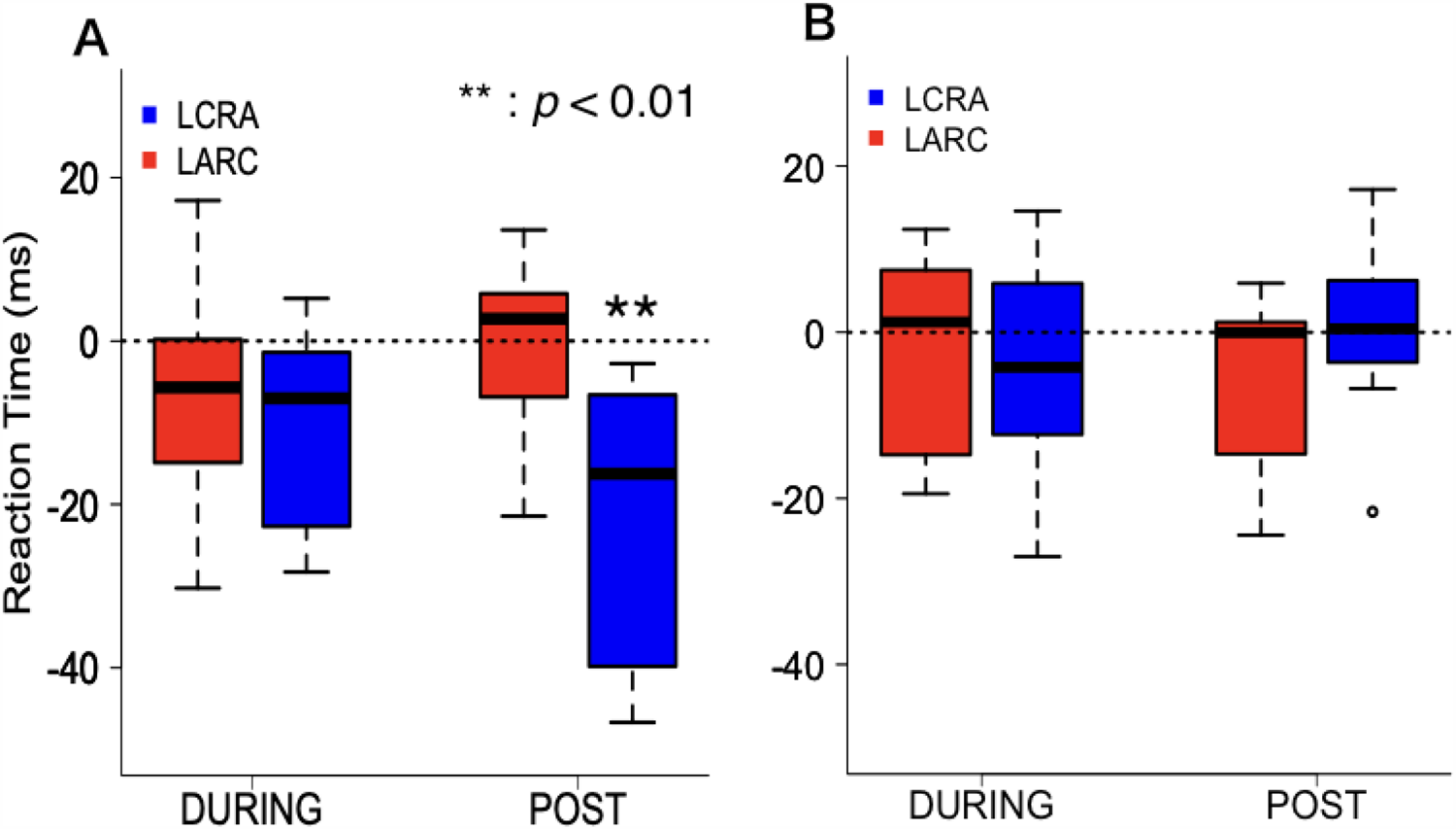
The change in reaction time on Day 1 (**A**) and Day 2 (**B**) relative to the PRE phase in each stimulation phase. Blue box, left cathode and right anode stimulation condition (LCRA); Red box, left anode and right cathode stimulation condition (LARC). The horizontal line inside each box represents the median, the boxes extend to the lower and upper quartiles, and the whiskers extend to the extreme values. Negative values indicate shortened reaction time, i.e. hand choice accelerated relative to the PRE phase.

Concerning the RT on day 1, two-way ANOVA revealed a significant interaction between the stimulation phases and conditions (*F* (2, 12) = 4.348, *p* = 0.038, *η*^2^_*p*_ = 0.42) and a main effect of stimulation phase (*F* (2, 12) = 5.047, *p* = 0.026, *η*^2^_*p*_ = 0.475) was significant. The main effect of stimulation conditions (*F* (1, 6) = 2.037, *p* = 0.203) was not significant. The simple main effect of stimulation phase under LCRA stimulation (*F* (2, 14) = 10.418, *p* = 0.002, *η*^2^_*p*_ = 0.598) was significant; however, it was not significant under LARC stimulation (*F* (2, 12) = 1.317, *p* = 0.304). Paired-sample multiple *t* tests corrected for multiple comparisons (Bonferroni correction) demonstrated that RT decreased significantly after LCRA stimulation relative to before the stimulation (*adj p* = 0.001, *d* = 1.72) (Fig. 8A). For RT on Day 2, two-way ANOVA revealed no significant interaction between the stimulation phase and condition (*F* (2, 12) = 0.632, *p* = 0.548) (Fig. 8B).

## Discussion

The present study demonstrated that the probability of left-hand choice increased and that of right-hand choice decreased significantly after stimulation under LCRA condition. A similar but marginally significant change in choice probability was also observed during the stimulation, thus, indicating that the decrease in the excitability of the left PPC and the increase in the excitability of the right PPC are essential to enhance left-hand choice. Our results are consistent with those of Oliveira et al.^4^; they reported that single-pulse TMS disruption of the left PPC just prior to the initiation of a reaching action increased the probability of left-hand use, which suggested the asymmetrical involvement of PPCs on hand choice. LTP or LTD of synapses are reportedly involved in the residual anode or cathode effects of tDCS, respectively^19–22^; such plastic changes in the synaptic transmission of the left and right PPCs by tDCS may underlie the observed influence of PPCs on hand choice.

We further considered the possibility that the left PPC is involved in planning the reaching actions of both hands and that the right PPC is involved in planning reaches for the left hand alone. A previous fMRI study that employed left- or right-hand reaching tasks observed that the activity of the contralateral (left) PPC significantly increased in comparison to that of the ipsilateral (right) PPC during right-hand reaches; in contrast, bilateral PPC activity increased during left-hand reaches^23^. As mentioned above, Fitzpatrick et al.^12^ measured the neural activity of the participants while they reached targets presented at random positions. Their bilateral PPC activity increased when they were allowed to choose the hand with which they reached for the target relative to when their hand-choice was mandated by instruction. The authors concluded that the both PPCs compete with each other and the hand contralateral to the hemisphere with relatively increased activity is selected. Our results were consistent with their PPIC model when the left PPC excitability was decreased and right PPC excitability was increased; specifically, this condition may directly excite left hand encodings in the right PPC while reducing the inhibition by the left PPC of left-hand encodings in the right PPC. The possible presence of left-hand encodings in the left PPC increases the likelihood of right-hand choice. However, this explanation does not extend to the decrease in the right PPC excitability and increase in the left PPC excitability because, as discussed by Fitzpatrick et al.^12^, right-handed individuals feature a strong right-hand bias. Right-hand choice is already the default, which leaves little potential to increase the probability of a right-hand choice. Further investigations in left-handed individuals are warranted to further test our hypothesis. It is also possible that the left and right PPC differentially encode left- and right-hand actions. More specifically, although Fitzpatrick et al.^12^ found bilateral PPC activation, encoding of the left and right hand in the PPC may be asymmetrical. Fumuro et al.^23^ reported that the dominant hand induces the activation of the contralateral hemisphere, while the non-dominant hand induces bilateral activation. Ipsilateral expression—the presence of left-hand encodings in the left PPC—may compensate for the suppression of left-hand encoding in the right PPC, thus, resulting in less effect on left hand choice when the excitability of the right PPC was decreased.

The target locations we focused on in RT analysis were situated near PSE where selection of the right and left hands competed. RT significantly shortened across LCRA stimulation condition on Day 1 but did not significantly change with LARC stimulation. These findings were similar to our observations regarding the changes in the hand-choice probability. The lack of a difference in RT on Day 2 may have been due to RT already having been sufficiently shortened for the participants to move more quickly. According to affordance competition model advanced by Cisek^24^, RT increases when competition between possible choices increases. Cisek^24^ reported that information collected from sight and proprioception as well as contextual information concerning rewards related to each selectable action is input to the PPC and PM, and the neural activity that encodes the action is finally selected after the body of evidence is considered; this process is referred to as the affordance competition model. Christopoulos et al.^25^ proposed a computational decision-making model that could also explain the extended RT in situations where selection is difficult due to extensive competition between the options. Oliveira et al.^4^ reported that RT extended more under TMS than in its absence. TMS administered to PPC added noise to the neural activity before choice selection, thus, preventing increased neural activity and prolonging the decision time. In contrast, in the present study, RT for hand choice was reduced by LCRA stimulation. The tDCS-mediated enhancement of neural activity in one hemisphere and tDCS-mediated suppression of neural activity in the other may have resulted in the acceleration of hand-choice decision-making on account of less competition in the neural activity. Cui et al.^11^ performed an experiment in which monkeys were required to either engage in saccades or reach for a target with their hands while their PPC activity was recorded. Neural activity related to both actions increased from target presentation to action choice; subsequently, the neural activity of the selected and discarded behaviours were retained and suppressed, respectively. These observations implicate the PPC in action selection as postulated by the affordance competition model. Javadi et al.^18^ reported that tDCS of the primary motor cortex can modulate hand choice during a perceptual decision-making task. Their results demonstrated that anodal stimulation of the primary motor cortex (PMC) biases participants’ responses towards using the contralateral hand whereas cathodal stimulation biases the responses towards the ipsilateral hand. This finding suggests that increase or decrease in PMC excitation were taken into consideration in the decision-making process in PPC where information about the left and right hand compete.

A limitation of this study is that we did not include sham conditions to test the placebo effect. However, we believe that the placebo effect has a limited influence on the conclusions of the study for multiple reasons. Firstly, the participants were instructed to reach the target as quickly and accurately as possible and were blinded to the purpose of the experiment, i.e. hand choice and stimulus polarities. Therefore, if a placebo effect existed, it would have affected the movement accuracy and speed more than hand choice. Secondly, all participants were right-handed and the results demonstrated an increase in left hand choice or no change. If the placebo effect affected the choice, it should have increased the right-hand choice to improve the performance. Lastly, the placebo effect would have similarly affected both LCRA and LARC conditions and would have less effect on the observed asymmetrical results between the conditions. Since the additional two sham conditions (LCRA sham and LARC sham) would have significantly increased the burden on the participants and deteriorated the quality of the data, we excluded them in the current study. To verify the effects of tDCS thoroughly, however, we believe that the sham conditions should be included in future studies. Another limitation was the simultaneous application of tDCS to the left and right PPC; this design prevented us from ascertaining whether stimulation of either hemisphere alone was sufficient to induce behavioural change. However, the reference electrode required in conventional tDCS cannot be placed at any location due to possible confounding effects in the experiment. We examined the possibility of placing a reference electrode on an extracranial location, such as the shoulder as reported by Ghanavati et al.^26^ However, since our task was the choice of hand, we cannot rule out the confounding effects of peripheral stimulation on one arm. Future works should employ high-definition tDCS, which enables refined localised stimulation, to stimulate the left and right PPCs independently and, thereby, ascertain whether a single area is more important in hand choice or whether simultaneous stimulation of both PPCs is essential. As mentioned above, our study was also limited by the inclusion of only right-handed individuals. Different data may have been observed in left-handed participants because of brain activity specific to the dominant hand^27^. The present study further observed individual differences in the effects of tDCS that may be attributable to individual differences between the participants in neural substrates or responses to tDCS administration. Indeed, internal factors such as the sex, age, circadian variation, genetic polymorphisms, and attentional function may affect the response to tDCS^28^. Another issue that should be noted is that 2-mA cathodal tDCS is reported to have an excitatory-enhancing effect rather than inhibitory effect when the stimulation was carried out for 20 min or longer^29,30^. With 5 min of stimulation, a significant inhibitory effect has been reported^31^, while with 15 min, the effect was not significant^30,32^. We believe that 10 min of stimulation can have an inhibitory effect or no effect but we cannot rule out the possibility of the excitatory-enhancing effect. If there was an excitatory-enhancing effect, the excitatory activation of both PPCs was the driving sources of the change in hand choice. Future confirmation of the effects of 2-mA 10-min cathodal stimulation is necessary. To solve this issue, monitoring of brain activity caused by tDCS stimulation using neuroimaging technology (EEG or functional Near-Infrared Spectroscopy) is required.

The results of the current study may be applied in the increase of paretic-hand use in patients with hemiparetic stroke whose quality of life is significantly diminished by stroke-induced difficulty in using paretic limbs effectively^33^. Although rehabilitation has been demonstrated to improve limb function to some extent, patients often only use their non-paretic limbs after discharge^34^. This learned non-use remains an unresolved issue in rehabilitative practice. The results of this study have demonstrated that 10 min of tDCS increased the probability of left-hand choice subsequently. Therefore, we recommend continued development of our methods and their application to promote the use of paretic upper limbs in the rehabilitation of patients with stroke.

## Conclusions

Stimulation of the right PPC with an anode and the left PPC with a cathode significantly increased the probability of left-hand choice and decreased RT. In contrast, stimulation of the right PPC with a cathode and the left PPC with an anode led to no significant change in either the choice or RT. Therefore, the present findings indicate that PPCs play an important and asymmetrical role in hand-choice control.

## Data Availability

The datasets generated and analysed during the current study are available from the corresponding author on reasonable request.
